# Allelic Spectra of Risk SNPs Are Different for Environment/Lifestyle Dependent versus Independent Diseases

**DOI:** 10.1371/journal.pgen.1005371

**Published:** 2015-07-22

**Authors:** Ivan P. Gorlov, Olga Y. Gorlova, Christopher I. Amos

**Affiliations:** The Geisel School of Medicine, Dartmouth College, Dartmouth-Hitchcock Medical Center, Lebanon, New Hampshire, United States of America; Georgia Institute of Technology, UNITED STATES

## Abstract

Genome-wide association studies (GWAS) have generated sufficient data to assess the role of selection in shaping allelic diversity of disease-associated SNPs. Negative selection against disease risk variants is expected to reduce their frequencies making them overrepresented in the group of minor (<50%) alleles. Indeed, we found that the overall proportion of risk alleles was higher among alleles with frequency <50% (minor alleles) compared to that in the group of major alleles. We hypothesized that negative selection may have different effects on environment (or lifestyle)-dependent versus environment (or lifestyle)-independent diseases. We used an environment/lifestyle index (ELI) to assess influence of environmental/lifestyle factors on disease etiology. ELI was defined as the number of publications mentioning “environment” or “lifestyle” AND disease per 1,000 disease-mentioning publications. We found that the frequency distributions of the risk alleles for the diseases with strong environmental/lifestyle components follow the distribution expected under a selectively neutral model, while frequency distributions of the risk alleles for the diseases with weak environmental/lifestyle influences is shifted to the lower values indicating effects of negative selection. We hypothesized that previously selectively neutral variants become risk alleles when environment changes. The hypothesis of ancestrally neutral, currently disadvantageous risk-associated alleles predicts that the distribution of risk alleles for the environment/lifestyle dependent diseases will follow a neutral model since natural selection has not had enough time to influence allele frequencies. The results of our analysis suggest that prediction of SNP functionality based on the level of evolutionary conservation may not be useful for SNPs associated with environment/lifestyle dependent diseases.

## Author Summary

We reviewed several thousand genome wide association studies that were conducted to identify genetic variants influencing risk of human diseases. We tested the hypothesis that single nucleotide polymorphisms (SNPs) that influence disease risk undergo positive or negative selection more frequently than an average SNP in the human genome. We found no evidence for excess of positive selection on disease-associated SNPs. At the same time we found that alleles associated with a higher disease risk undergo negative selection. We also demonstrated that risk alleles for diseases with strong influence of environment/lifestyle factors (e.g. Type II diabetes) show little evidence of negative selection, while risk alleles for diseases with weak influence of environment/lifestyle factors (e.g. Pathological myopia) show clear signs of negative selection. The approach used in this study can be used to estimate the number of genetic variants in the human genome influencing risk of human diseases.

## Introduction

Diseases play a central role in human evolution, influencing population frequencies of genetic polymorphisms directly or indirectly through hitchhiking or bottle neck effect [1–3]. Nonetheless, the role of selection in the shaping of population frequencies of the genetic variants associated with risk of common human diseases is poorly understood.

### Environment/lifestyle diseases

Both environmental and genetic factors influence risk of common human diseases, however, the relative significance of genetic and environmental factors in disease etiology differs for different diseases. A number of common human diseases including cardiovascular diseases and type 2 diabetes are believed to predominantly result from changes in lifestyle and environment [4,5] Having environment or lifestyle as a major risk factor does not rule out an influence of genetic polymorphisms. An assessment of the effects of selection on the risk alleles of the common diseases stratified by the importance of the environmental/lifestyle component has never been conducted before.

### Selection and disease associated SNPs

A number of methods to detect signatures of recent positive selection have been proposed, including Tajima’s D [6,7], selective sweep [8], tests based on fixation index used as a measure of population differentiation [9], haplotype analysis [10], tests based on the ratio of nonsynonymous and synonymous substitutions [11], and others [12–15]. The aforementioned methods work well when adaptation is driven by a single polymorphic locus (monogenic model); however, in the situation when adaptation is driven by multiple loci (polygenic model) selection may not produce the classical signature of selective sweep [16], see also [17] and [18]. Fixation of a beneficial mutation is also strongly affected by temporal variation in population size and selection pressure [19].

Some studies suggest that SNPs with the signature of recent positive selection tag regions associated with common human diseases [20]. Raj et al. 2013 [21] found that several loci linked to the risk of inflammatory diseases carry genomic signatures of recent positive selection. It also has been demonstrated that SNPs associated with the risk of type II diabetes carry signature of recent positive selection [22]. Then again, other studies found no evidence of positive selection at loci linked to common human diseases [23,24].

Unfortunately, the cited studies do not provide an answer to the important question whether the SNPs with a signature of recent positive selection have higher likelihood to be detected (and reported) as disease-associated compared to those without such signature. Wang and Pike [25] suggested that allelic spectra of SNPs associated with common diseases should be similar to the allelic spectra for the entire human genome (which basically follow neutral model). They built their hypothesis based on the fact that the number of disease loci for common disease is usually high and each locus makes only a minor contribution to a disease. They argue that natural selection has been operating weakly and for a short time, suggesting that the majority of SNPs associated with common disease may be near-neutral.

We and others hypothesized that disease-associated SNPs experience negative selection [26–30]. Detection of negative selection is more challenging than detection of recent positive selection because it does not reshape genetic variation in selected region. The main indicator of negative selection is deviation of allelic frequencies from the distribution expected under the neutrality model towards lower values [28,31]. Lower minor allele frequency expected as a result of negative selection cannot be estimated for individual SNPs but only for SNP classes, e.g. nonsynonymous or disease risk-associated SNPs. Even though it has been shown that disease associated SNPs tend to occur in evolutionary conserved regions [32] the effect of negative selection on disease risk associated SNPs is poorly understood.

### Genome-wide association studies (GWAS)

Genome-wide association studies are widely used to identify SNPs associated with risk of common diseases. Thousands GWASs have been conducted with the results reported in several databases. One of the most comprehensive databases is the catalogue of published GWASs (CPGWAS) [33] (http://www.genome.gov/gwastudies/). More than 7,000 SNPs linked to nearly 5,000 genes have been reported in CPGWAS making it a valuable resource to study the role of natural selection in the shaping of genetic variation of common human disease.

The goal of our study was to evaluate the effect of positive and negative selection on allelic spectra of SNPs associated with the risk of common human diseases and to assess how allelic spectra differ for environment/lifestyle dependent versus environment/lifestyle independent diseases.

## Results and Discussion

### The number of SNPs with a signature of recent positive selection on commonly used genotyping platforms

**Fig 1:**
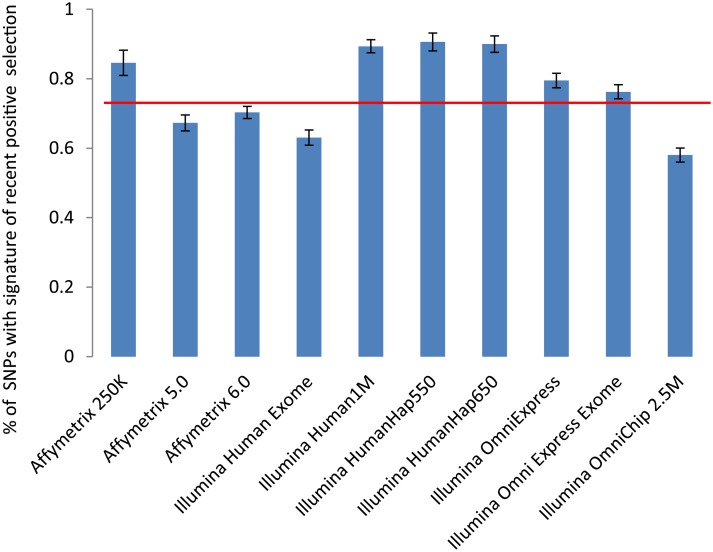
Proportions of SNPs with the signature of recent positive selection on the most commonly used genotyping platforms. The red horizontal line shows the proportion of SNPs with signature of recent positive selection among GWAS-detected SNPs associated with risk of common human diseases.

Fig 1 shows the proportions of the SNPs with the signature of recent positive selection across commonly used genotyping platforms. The lowest proportion was on Illumina OmniChip 2.5M platform—0.58%, and the highest proportion was on Illumina Human Hap550 platform—0.91%. The average proportion of SNPs with the signature of recent positive selection across all genotyping platforms was essentially the same as the proportion of SNPs with the signature of recent positive selection among GWAS-detected disease associated SNPs reported in CPGWAS—0.75%±0.09% versus 0.76%±0.17% (*x* ^2^ = 0.09, df = 1, P = 0.95). A comparison of the proportion of SNPs with the signature of recent positive selection among CPGWAS-reported disease-associated SNPs with proportions of the SNPs on individual platforms demonstrated that it was significantly higher for Illumina 1M platform (P = 0.03) and lower for OmniChip 2.5M (P = 0.02). The differences, however, became statistically non-significant after multiple testing adjustments. Table 1 shows details of the estimation of the proportions of SNPs with the signature of recent positive selection on the 10 most commonly used genotyping platforms.

**Table 1:** Absolute number and the proportion of selected SNPs on the most popular genotyping platforms.

Genotyping Platform	# of SNPs	SNPs with evidence of positive selection (EPS)	% SNPs with EPS	SE*
Affymetrix 250K	250,000	2,115	0.85	0.02
Affymetrix 5.0	500,500	3,368	0.67	0.01
Affymetrix 6.0	906,600	6,376	0.70	0.01
Human Exome 12v1	240,000	314	0.63	0.01
Illumina Human1M	1,000,000	8,934	0.89	0.01
Illumina HumanHap550	550,000	4,984	0.91	0.01
Illumina HumanHap650	650,000	5,849	0.90	0.01
Illumina OmniExpress	730,500	5,808	0.80	0.01
Illumina Omni Express Exome	730,500	5,571	0.76	0.01
Illumina OmniChip 2.5M	2,015,000	5,650	0.58	0.01

* SE—Standard Error

Therefore we found that among GWAS detected SNPs the proportion of SNPs with evidence of recent positive selection is the same as the proportion of the SNPs on genotyping platforms. This result suggests that recent positive selection does not increase (or decrease) the chance that a SNP will be reported as disease-risk associated. This also suggests that reported evidence of positive selection on disease risk associated SNPs [21,34,35] may largely result from simple random overlap between disease associated and positively selected SNPs.

### Minor risk alleles (MiRA)

Minor Risk Alleles (MiRA) were defined as risk-associated alleles with frequency less than 50%. We used MiRA proportion as an estimator of the effect of negative selection on allelic frequencies. If the probability to be risk-associated or protective does not depend on allelic frequency, the expected MiRA proportion will be 0.5. Table 2 shows MiRA proportions for the diseases with at least 20 CPGWAS reported risk associated SNPs. Analyses of Variance (ANOVA) show significant variation of the MiRA proportion among common diseases: F = 2.3, df = 24, P = 0.000001. The MiRA proportions vary from 0.45±0.1 for Graves' disease to 0.96±0.04 for chronic kidney disease.

**Table 2:** Proportions of minor risk alleles (MiRA) in GWAS studied diseases.

Disease	MiRA	# of SNPs	SE
Crohn's disease	0.61	151	0.04
Rheumatoid arthritis	0.68	120	0.04
Inflammatory bowel disease	0.50	116	0.05
Breast cancer	0.64	85	0.05
Type 2 diabetes	0.51	79	0.06
Ulcerative colitis	0.56	77	0.06
Prostate cancer	0.63	71	0.06
Coronary heart disease	0.61	56	0.07
Obesity	0.55	55	0.07
Systemic lupus erythematosus	0.71	55	0.06
Schizophrenia	0.47	47	0.07
Age-related macular degeneration	0.52	42	0.08
Myopia (pathological)	0.72	51	0.07
Type 1 diabetes	0.55	38	0.08
Alzheimer's disease (late onset)	0.58	36	0.08
Parkinson's disease	0.55	33	0.09
Colorectal cancer	0.66	32	0.09
Celiac disease	0.59	32	0.09
Asthma	0.58	31	0.09
Psoriasis	0.61	28	0.09
Chronic lymphocytic leukemia	0.54	28	0.10
Chronic kidney disease	0.96	27	0.04
Primary biliary cirrhosis	0.63	27	0.09
Multiple sclerosis	0.60	25	0.10
Graves' disease	0.45	22	0.11

There is a considerable heterogeneity between diseases by GWAS sample sizes. A larger sample size translates into a higher statistical power to detect SNPs with a low minor allele frequency (MAF). However it is unlikely that the sample size will influence the probability that a minor allele will be associated with risk rather than protection. Consistent with this expectation we found that larger studies were more likely to detect rare (MAF≤0.05) SNPs (Spearman rank order correlation = 0.14, N = 1,657, P = 0.00002). However, no association was found between the sample size and direction of the effect of minor alleles (MiRA) (Spearman rank order correlation = -0.04, N = 1,657, P = 0.56).

### Environment/lifestyle index (ELI)

Text mining is a powerful tool to infer the relationships between diverse biological entities [36,37]. We used it to assess the role of environment/lifestyle factors in disease etiology. We cannot simply search for publications linking disease to environmental or lifestyle factors because we will find such publications for any human disease. A more objective measure of influences of environment/lifestyle factors on disease etiology is needed. It is reasonable to suggest that the proportion of papers simultaneously referring to a disease and environment/lifestyle factors will be higher for diseases with strong environment/lifestyle influences. Table 3 shows estimated environment/lifestyle indices (ELIs) for the diseases that were targeted by at least 3 independent GWASs. The highest ELIs were detected for obesity and type II diabetes, 112.8 and 76.4 correspondingly, and the lowest for pancreatic cancer and primary biliary cirrhosis, 10.9 and 9.6 correspondingly.

**Table 3:** Environmental and lifestyle indexes (ELIs) for the GWAS-studied diseases.

Disease	N of publications containing:			ELI
	Disease Name (DN)	DN AND "environment"	DN AND "lifestyle"	
Obesity	210416	7892	15834	112.8
Type 2 diabetes	113333	2180	6477	76.4
Major depressive disorder	96868	3626	3466	73.2
Asthma	147762	9239	1152	70.3
Atopic dermatitis	19705	1100	144	63.1
Autism	28552	1437	98	53.8
Attention deficit hyperactivity disorder	25208	1160	164	52.5
Melanoma	98871	4546	237	48.4
Schizophrenia	112478	4004	949	44.0
Bipolar disorder	38944	1016	524	39.5
Coronary heart disease	262453	4267	5647	37.8
Psoriasis	37149	1208	167	37.0
Type 1 diabetes	68482	1561	875	35.6
Alzheimer's disease (late onset)	2958	56	35	30.8
Multiple sclerosis	63493	1565	331	29.9
Age-related macular degeneration	21515	506	111	28.7
Migraine	29512	569	264	28.2
Sudden cardiac arrest	25821	487	229	27.7
Breast cancer	292315	4727	2742	25.6
Migraine with aura	4446	89	23	25.2
Amyotrophic lateral sclerosis	18004	394	49	24.6
Amyotrophic lateral sclerosis (sporadic)	18004	394	49	24.6
Inflammatory bowel disease	74179	1425	351	23.9
Prostate cancer	123622	1855	1040	23.4
Colorectal cancer	172036	2470	1356	22.2
Crohn's disease	41342	732	178	22.0
Ulcerative colitis	36344	600	168	21.1
Parkinson's disease	76837	1368	248	21.0
Lung cancer	245974	4112	916	20.4
Myopia (pathological)	3990	77	2	19.8
Chronic kidney disease	117185	1281	961	19.1
Endometriosis	21212	342	59	18.9
Bladder cancer	62487	884	289	18.8
Systemic sclerosis	22938	387	36	18.4
Systemic lupus erythematosus	58576	929	110	17.7
Rheumatoid arthritis	120346	1578	393	16.4
Testicular germ cell tumor	27123	313	99	15.2
Ovarian cancer	86537	888	353	14.3
Graves' disease	18020	196	45	13.4
Celiac disease	20718	213	55	12.9
Acute lymphoblastic leukemia	32530	335	57	12.1
Chronic lymphocytic leukemia	19285	209	23	12.0
Pancreatic cancer	73158	622	172	10.9
Primary biliary cirrhosis	12347	93	25	9.6

To test stability of ELI-based ranking of human diseases we extended the ELI by including the additional term: “exposure”. S1 Table shows disease ranking based on ELI and extended ELI. In the ranking based on the extended ELI, lung cancer moved from 29^th^ position to 9^th^ following obesity, asthma, atopic dermatitis, type 2 diabetes, major depressive disorder, melanoma, autism, and attention deficit hyperactivity disorder. Overall disease ranking was similar for ELI and extended ELI (S1 Fig). Out of the top 10 ELI-defined environment/lifestyle dependent diseases all except “bipolar disorder” are also among the top 10 environment/lifestyle dependent diseases defined based on the extended ELI. The correlation coefficient between ELI and extended ELI was 0.92, N = 44, P = 1.6x10^-17^. Replacing ELI by extended ELI for disease ranking did not change our conclusions. Analysis of the extended ELI demonstrates that (i) the ELI-based approach for identification of environment/lifestyle dependent disease is not perfect and can rank some diseases (such as lung cancer) lower than we may think is accurate, and (ii) overall ELI based classification provides a sufficiently accurate and robust assessment of environmental and lifestyle related effects on disease risk to capture most known influences on disease risks. Analysis of the human disease with extended ELI suggests that the proposed classification works effectively on the large collection of diseases used in this analysis.

### ELI and risk allele frequencies

**Fig 2:**
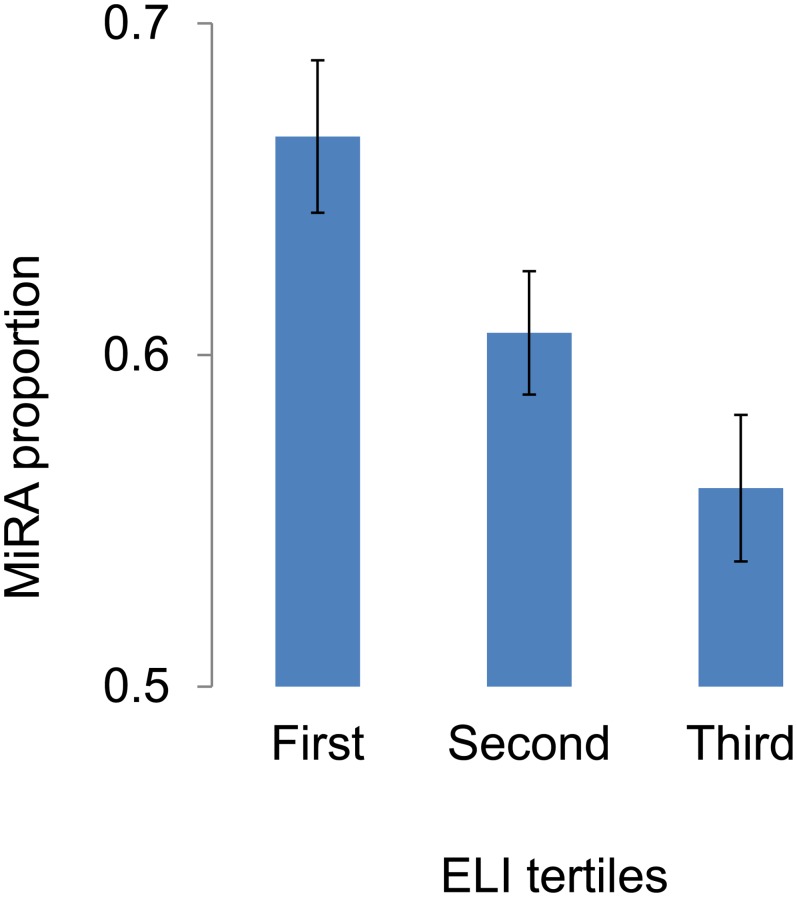
The proportions of minor risk alleles (MiRA) in the first, second and third tertiles defined based on the environment/lifestyle index (ELI).

We found that risk allele frequencies were higher for environment/lifestyle dependent diseases: Spearman rank order correlation coefficient between risk allele frequencies and ELI was 0.1 (P = 0.0002). We further subdivided diseases in tertiles based on ELI and estimated MiRA proportions in each tertile (Fig 2). There was a significant variation among tertiles by the MiRA proportions: ANOVA-test F = 5.02, df = 2, P = 0.007. The proportion was highest in the first and lowest in the third tertile. The analysis indicates that the risk alleles for environment/lifestyle dependent diseases tend to be more common compared to the risk alleles for environment/lifestyle independent diseases.

We additionally performed nonparametric Spearman rank order correlation analysis. Significant positive association between risk allele frequency and ELI was detected (Spearman R = 0.12, N = 1547, P = 4 x 10^−6^). This result supports the conclusion that risk alleles for environment/lifestyle dependent diseases tend to have a higher frequency compared to the risk alleles for environment/lifestyle independent diseases.

**Fig 3:**
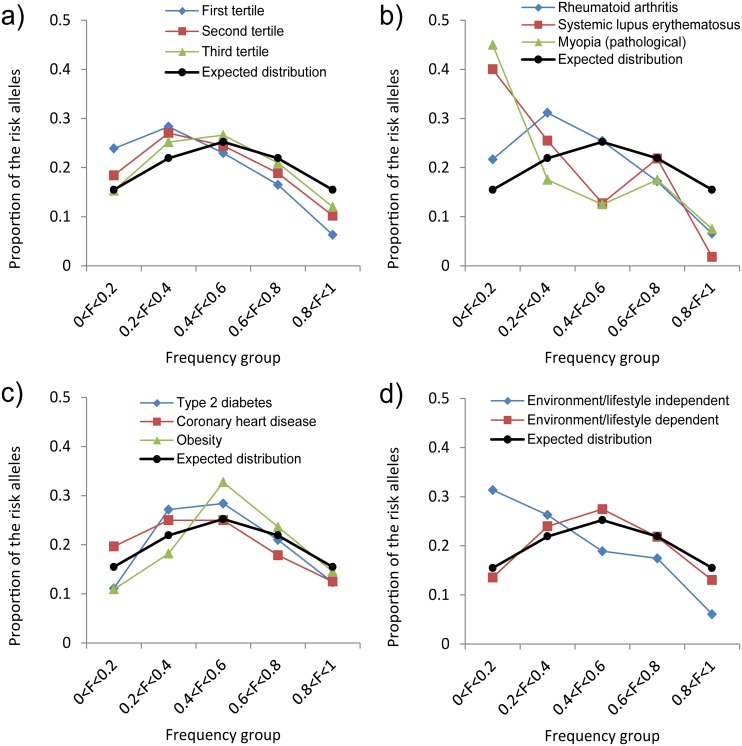
Frequency binned distributions of the risk alleles for different common diseases. F—frequency of the risk-associated allele. Area under each curve equals 1. Black line shows the distribution expected under the assumption that the probability of the allele to be risk associated is independent of its frequency. a) Proportions of the risk alleles in the 5 frequency categories for diseases stratified by the ELI tertiles. b) Proportions of the risk alleles for 3 individual diseases from the first tertile (environment/lifestyle independent diseases). c) Distributions of risk alleles for 3 individual diseases from the third ELI tertile (environment/lifestyle dependent diseases). d) Proportions of the risk alleles averaged for the 3 environment/lifestyle dependent (red line) and 3 environment/lifestyle independent (blue line) diseases.

To get a more detailed picture of the association between risk allele frequency and disease dependences on environmental/lifestyle factors we assessed risk allele frequency distributions for the diseases from the first, second and third ELI tertiles (Fig 3a). The diseases were selected based on the condition that they have at least 50 reported risk associated SNPs to allow a reliable estimation of the frequency distribution. We took 3 individual diseases from the first tertile (rheumatoid arthritis, systemic lupus erythematosus and pathological myopia) and compared them to 3 environment/lifestyle dependent diseases—those from the third tertile (type 2 diabetes, coronary heart disease and obesity). The distributions of the risk allele frequencies for environment/lifestyle independent diseases were asymmetrical and shifted to the left, indicating effect of negative selection (Fig 3b). The distributions of the risk allele frequencies for environment/lifestyle dependent diseases were more symmetrical indicating a weak influence of negative selection (Fig 3c). The differences between environment/lifestyle dependent and independent diseases were more evident when we compared the proportions of risk alleles averaged across diseases (Fig 3d). The distribution of the risk alleles for environment/lifestyle independent diseases was shifted towards a predominance of rarer SNPs while the distribution of proportions of the risk alleles for environmental lifestyle dependent diseases was almost perfectly symmetrical and bell-shaped.

Common human diseases may create conditions for positive selection for disease protective alleles, while risk associated alleles are expected to be slightly deleterious [26,27,30] and therefore to be under pressure of negative selection. Based on these considerations we expected that GWAS detected disease-associated SNPs will show signals of positive and/or negative selection. We found, however, that the proportion of GWAS-detected SNPs with the signature of recent positive selection does not differ from the proportion of SNPs with the signature of recent positive selection on genotyping platforms, suggesting that disease associated SNP have the same chances to be positively selected during the process of GWAS analysis as an average SNP in the human genome. On the other hand, our analysis supports the hypothesis that risk-associated alleles frequently undergo negative selection. We found that risk-associated alleles are more common among minor alleles. The overall distribution of the risk alleles is shifted to lower frequencies indicating an effect of negative selection against risk-associated variants. We further hypothesized that the effects of negative selection on allelic spectra may be different for environment/lifestyle dependent versus environment/lifestyle independent diseases. We found environment/lifestyle dependent diseases tend to have a higher frequency of the risk associated variants suggesting a weaker effect of negative selection.

It is widely accepted that the majority of genetic variants in human populations are neutral [38,39]. It is also known that selective value of the variants depends on the environment [40,41]. A neutral variant may become advantageous (or disadvantageous) when environment changes. For example, mutations controlling lactose tolerance were initially neutral and became advantageous about 5,000–8,000 BC, after domestication of cattle [42,43]. It is becoming more and more evident that many common human diseases are caused by changes in environment and/or lifestyle [44–48]. Changes in environment or lifestyle may redefine functional significance of existing neutral SNPs. One can expect that the majority of risk associated variants for environment/lifestyle dependent diseases are recently recruited from the pool of selectively neutral variants. Whether those formerly neutral variants will be risk-associated or disease protective depends on how they influence biology. It is unlikely that direction of the effect (risk-associated or protective) of a recently neutral variant will depend on its frequency. Let’s assume, for example, that there is a SNP that slightly modulates the expression level of some gene and its effect is selectively neutral. In this case the frequency of the allele associated with a low expression level is not influenced by selection, so this variant can be minor (<50%) or major (>50%). Let’s assume that changes in environment or lifestyle made a low expression level of the gene associated with increased risk. In this scenario the distribution of the risk-associated alleles will initially follow the neutral model even though it is not selectively neutral anymore. It will take time (tens to hundreds generations, depending on the selective pressure) for the negative selection to reduce the frequency of the risk alleles.

**Fig 4:**
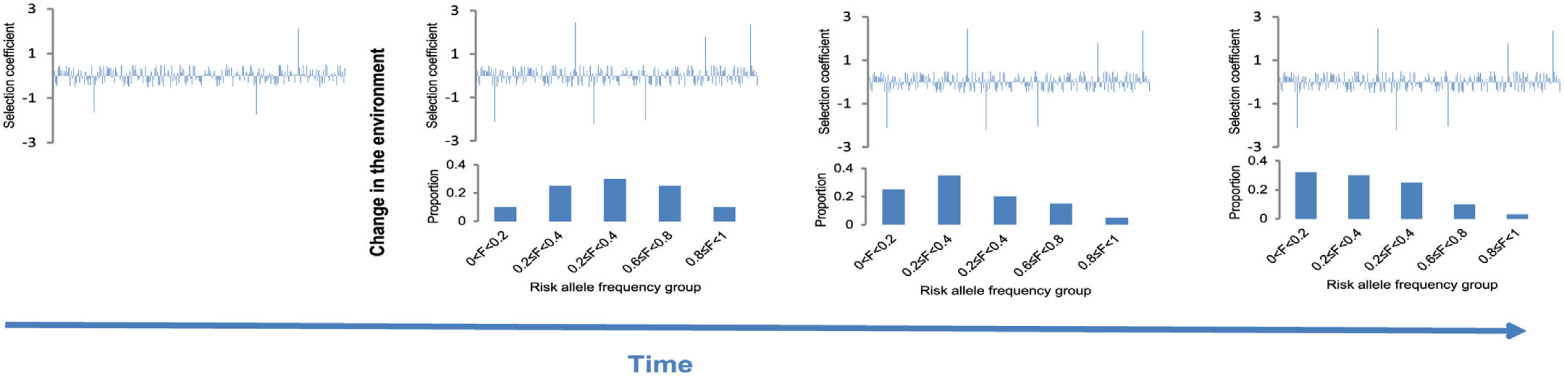
Expected evolutionary dynamics of currently deleterious, recently neutral risk associated alleles. Upper panel shows the distribution of selection coefficients: negative values imply negative and positive values positive selection. A change in the environment or life style leads to changes in selective values of existing variants making some of previously neutral variants deleterious and others advantageous. The lower panel shows frequency distributions of risk alleles immediately after changes in environment/life style and after the negative selection took place.

Therefore, even though many formally neutral risk variants are (currently) deleterious, their allelic spectra will follow the neutral model for some time. Fig 4 depicts the hypothesis of recently neutral, currently deleterious risk-associated variants. The figure shows a hypothetical example with individual selection coefficients (upper panel) and frequency distributions of the risk alleles (lower panel). According to the proposed model, changing environment reassigns selective values of existing SNPs which is indicated by different profile of selection coefficients (upper panel) before and after the change in the environment took place. Immediately after changes in the environment the frequency distribution of risk associated alleles is symmetrical. Negative selection against risk alleles reduces their frequencies, shifting the distribution to the left and making it asymmetrical.

Environmental diseases are defined as diseases whose incidence can be directly related to effects of environmental factors. Disease-causing environmental factors include but are not limited to stress, physical and mental abuse, diet, exposure to toxins, pathogens, radiation, and chemicals. Many common human diseases are considered to be environmental [49–51]. In the context of this study by environment/lifestyle factors we mean recent (less than several generations away) changes in lifestyle and environment. Such changes redefine selective profiles on existing SNPs, but because they are recent, there is not sufficient time for selection to change allelic frequencies.

Based on the results of our analysis, risk-associated alleles can be roughly divided into two categories: evolutionarily old and evolutionarily young. Old alleles have a long history of being risk associated so natural selection has had enough time to influence their frequencies. Young risk alleles recently came from the pool of selectively neutral variants and because of that history, selection has not had sufficient time to influence their frequencies. One can expect that alleles associated with the risk of environment/lifestyle dependent diseases will most often be young whereas the alleles associated with the risk of environment/lifestyle independent diseases will more often be evolutionarily old. The proportions of young and old alleles for a given disease can be roughly estimated by comparing the frequency distribution of risk variants with the distribution expected under the null, under which the probability to be risk associated is frequency independent. Currently the frequency distribution of risk variants can be reasonably estimated for a limited number of well-studied diseases only, but with the advance of GWASs this information will be available for more and more diseases.

The hypothesis of recently neutral, currently disadvantageous risk-associated alleles has several practical implications. First of all, recently neutral, currently deleterious alleles do not carry a signature of positive or negative selection which makes the prediction of their functionality based on the level of evolutionary conservation questionable. Besides, because frequency spectra of the risk-associated variants follow the neutral model, one may predict the number of risk-associated variants in different frequency groups (under the neutral model we assume that the effect size is independent of allelic frequency) which can be used to estimate the sample size required for the detection of SNPs from a specified frequency range. The results of our analysis suggest that the nearly-neutral model is applicable to common disease variants resulting from recent changes in environment and/or life style which convert neutral variants into slightly deleterious (risk associated) or advantageous (protective).

## Materials and Methods

### Disease-associated SNPs

SNPs associated with the risk of human diseases were retrieved from the Catalogue of Published Genome-Wide Association Studies (CPGWAS) (http://www.genome.gov/26525384/)[33]. The CPGWAS was accessed on December 15, 2014. SNPs with reported P-values of 5·10^−8^ or lower were used in the analysis.

### SNPs with a signature of recent positive selection

All tests for detecting the signature of recent positive selection are quantitative and a decision is made based on specified thresholds [12,52]. As a result, the lists of the SNPs with the signature of recent positive selection vary depending on the method and thresholds chosen. We used 24,060 SNPs with the signature of recent positive selection reported in the database of positive selection in human populations (dbPSHP)[53]. Those SNPs were identified by applying a set of stringent filters that are consistent across 6 most commonly used approaches to detect a signature of recent positive selection: Tajima’s D, Integrated Haplotype Score, Extended Haplotype Homozygosity, Cross-Population Composite Likelihood Ratio, Difference of Derived Allele Frequency, and Fixation Index [53].

### Assessing effects of negative selection on allelic spectra

Both minor alleles (those with the frequency < 50%) and major alleles (those with the frequency >50%) can be risk associated—risk alleles. The frequency distribution which includes frequencies of both minor and major alleles of the SNPs by definition will be symmetrical, since the absolute majority of the SNPs (more than 95%) are biallelic with one minor and one reciprocal major allele. If a minor allele has the same chance to be risk-associated as the reciprocal major allele, the frequency distribution of the risk alleles should be symmetrical.

Note that overall distribution of SNP’s allele frequencies is symmetrical and U-shaped [54]. This is because proportion of rare SNPs in the human genome is higher than proportion of common SNPs. Distribution of the GWAS-detected disease-associated SNPs is bell-shaped because common SNPs are overrepresented on genotyping platforms and also because GWASs are underpowered to detect rare disease-associated SNPs.

Negative selection against risk-associated variants will increase the proportion of the risk-associated variants among alleles with minor frequency. We used the proportion of the risk alleles with minor allele frequencies—minor risk alleles (MiRA) as an estimator of the effect of negative selection. Under the null hypothesis—rare (minor) alleles have the same chances to be risk associated as the reciprocal common (major) allele—MiRA proportion is expected to be 0.5. The stronger the negative selection against the risk-associated variant, the higher MiRA proportion will be.

We also assessed the distributions of the risk-associated alleles by their frequencies. Risk alleles were binned into 5 frequency (F) groups: 0<F<0.2, 0.2≤F<0.4, 0.4≤F<0.6, 0.6≤F<0.8, 0.8≤F<1. We chose 5 groups because it is optimal for the available sample sizes. Data on the population frequency of the risk alleles were from original GWASs. We used reported frequencies of the risk-associated alleles in controls.

We did not use ancestral/derived allele status in the analysis even though it has been shown to be relevant to selection and risk of common diseases [55,56]. The reason for this was that ancestral/derived status is not available for many SNPs, especially those located in intronic or intergenic regions. Using ancestral information in this analysis would have reduced the number of SNPs we could evaluate and introduce a bias because SNPs would have been excluded from analysis based on the level of evolutionary conservation (ancestral/derived status information is only available for SNPs located in evolutionary conserved regions, which allows sequence alignment from multiple species [57]).

### Environment/lifestyle index (ELI)

Genetic share of the disease risk can be assessed by disease heritability. Estimated disease heritability varies from less than 5% for stomach cancer [58] to almost 90% for type 1 diabetes [59]. Unfortunately estimates of the disease heritability are not reliable [60] and can be confounded by shared environment [61,62].

We applied text mining to estimate relative influence of environmental and lifestyle factors on disease etiology. Environment/Lifestyle Index (ELI) was used as a measure of the influence of environmental and lifestyle factors on disease etiology. To estimate ELI we first searched PubMed for the disease name, e.g. “rheumatoid arthritis”, and identified papers with disease name in the abstract. Next we identified the number of papers mentioning together disease name and “environment” or “lifestyle”. ELI was computed as the number of the papers mentioning disease name AND environment or lifestyle per 1,000 papers mentioning disease name. As an example, there are 120,346 abstracts mentioning rheumatoid arthritis, 1,578 abstracts mentioning “rheumatoid arthritis” and “environment”, and 393 abstracts mentions “rheumatoid arthritis” and “lifestyle” which give ELI for rheumatoid arthritis: ELI_RI_ = (1,578+393)/120,346*1000 = 16.4.

Analysis of the temporal dynamics of disease prevalence might be useful in identification of diseases influenced by recent changes in environment and lifestyle. Unfortunately information on disease prevalence is not available for many diseases, especially concerning the temporal dynamics in disease prevalence. This was the reason that we used environment/lifestyle index rather than disease prevalence as a measure of disease dependence of environment/lifestyle.

### Commonly used genotyping platforms

The list of 10 most commonly used genotyping platforms was obtained by reviewing platforms listed on CPGWAS database. For each platform we retrieved the list of SNPs using manufacturers’ data and among them identified SNPs with the signature of recent positive selection from dbPSHP database [53].

### Statistical analysis

Statistical analysis was done using STATA software (version 10, StataCorp LP, College Station, TX). We used *x* ^2^ test to compare observed to expected proportions. We applied nonparametric statistical tests, e.g. Spearman rank test, for the datasets with significant deviation from normal distribution.

## Supporting Information

S1 Table Extended environment/lifestyle index. (DOCX)

S1 Fig Environment/lifestyle index (ELI) versus extended environment/lifestyle index (EELI). (DOCX)
