# Supplementary material for: Allelic Spectra of Risk SNPs Are Different for Environment/Lifestyle Dependent versus Independent Diseases
Source: PLoS Genet. 2015 Jul 22;11(7):e1005371. doi: 10.1371/journal.pgen.1005371 (PMC4511800; doi:10.1371/journal.pgen.1005371)
Supplement: S1 Table — (DOCX) [file pgen.1005371.s001.docx]

**Supplementary Materials**

**Table s1.** Extended Environment/Lifestyle Index

| Disease | Number of publications containing: | | | | ELI | Extended  ELI | ELI  ranking | Extended ELI ranking |
| --- | --- | --- | --- | --- | --- | --- | --- | --- |
|  | Disease Name  (DN) | DN AND  "Environment" | DN AND  "Lifestyle" | DN AND  "Exposure" |  |  |  |  |
| Obesity | 210416 | 7892 | 15834 | 5765 | 112.8 | 180.2 | 1 | 1 |
| Asthma | 147762 | 9239 | 1152 | 14486 | 70.3 | 168.4 | 4 | 2 |
| Atopic dermatitis | 19705 | 1100 | 144 | 1325 | 63.1 | 130.4 | 5 | 3 |
| Type 2 diabetes | 113333 | 2180 | 6477 | 2627 | 76.4 | 120.7 | 2 | 4 |
| Major depressive disorder | 96868 | 3626 | 3466 | 2424 | 73.2 | 108.2 | 3 | 5 |
| Melanoma | 98871 | 4546 | 237 | 4646 | 48.4 | 95.4 | 8 | 6 |
| Autism | 28552 | 1437 | 98 | 1057 | 53.8 | 90.8 | 6 | 7 |
| Attention deficit hyperactivity disorder | 25208 | 1160 | 164 | 958 | 52.5 | 90.5 | 7 | 8 |
| Lung cancer | 245974 | 4112 | 916 | 14039 | 20.4 | 77.5 | 29 | 9 |
| Schizophrenia | 112478 | 4004 | 949 | 2384 | 44.0 | 65.2 | 9 | 10 |
| Bladder cancer | 62487 | 884 | 289 | 2883 | 18.8 | 64.9 | 33 | 11 |
| Psoriasis | 37149 | 1208 | 167 | 881 | 37.0 | 60.7 | 12 | 12 |
| Breast cancer | 292315 | 4727 | 2742 | 9196 | 25.6 | 57.0 | 19 | 13 |
| Bipolar disorder | 38944 | 1016 | 524 | 676 | 39.5 | 56.9 | 10 | 14 |
| Age-related macular degeneration | 21515 | 506 | 111 | 593 | 28.7 | 56.2 | 16 | 15 |
| Amyotrophic lateral sclerosis | 18004 | 394 | 49 | 550 | 24.6 | 55.2 | 21 | 16 |
| Amyotrophic lateral sclerosis (sporadic) | 18004 | 394 | 49 | 550 | 24.6 | 55.2 | 22 | 17 |
| Parkinson's disease | 76837 | 1368 | 248 | 2552 | 21.0 | 54.2 | 28 | 18 |
| Type 1 diabetes | 68482 | 1561 | 875 | 1208 | 35.6 | 53.2 | 13 | 19 |
| Coronary heart disease | 262453 | 4267 | 5647 | 3535 | 37.8 | 51.2 | 11 | 20 |
| Multiple sclerosis | 63493 | 1565 | 331 | 1295 | 29.9 | 50.3 | 15 | 21 |
| Testicular germ cell tumor | 27123 | 313 | 99 | 946 | 15.2 | 50.1 | 37 | 22 |
| Prostate cancer | 123622 | 1855 | 1040 | 3178 | 23.4 | 49.1 | 24 | 23 |
| Alzheimer's disease (late onset) | 2958 | 56 | 35 | 44 | 30.8 | 45.6 | 14 | 24 |
| Colorectal cancer | 172036 | 2470 | 1356 | 3917 | 22.2 | 45.0 | 25 | 25 |
| Acute lymphoblastic leukemia | 32530 | 335 | 57 | 1040 | 12.1 | 44.0 | 41 | 26 |
| Inflammatory bowel disease | 74179 | 1425 | 351 | 1446 | 23.9 | 43.4 | 23 | 27 |
| Systemic sclerosis | 22938 | 387 | 36 | 559 | 18.4 | 42.8 | 34 | 28 |
| Crohn's disease | 41342 | 732 | 178 | 845 | 22.0 | 42.5 | 26 | 29 |
| Ovarian cancer | 86537 | 888 | 353 | 2418 | 14.3 | 42.3 | 38 | 30 |
| Migraine | 29512 | 569 | 264 | 392 | 28.2 | 41.5 | 17 | 31 |
| Chronic lymphocytic leukemia | 19285 | 209 | 23 | 548 | 12.0 | 40.4 | 42 | 32 |
| Sudden cardiac arrest | 25821 | 487 | 229 | 319 | 27.7 | 40.1 | 18 | 33 |
| Chronic kidney disease | 117185 | 1281 | 961 | 2199 | 19.1 | 37.9 | 31 | 34 |
| Endometriosis | 21212 | 342 | 59 | 378 | 18.9 | 36.7 | 32 | 35 |
| Ulcerative colitis | 36344 | 600 | 168 | 560 | 21.1 | 36.5 | 27 | 36 |
| Systemic lupus erythematosus | 58576 | 929 | 110 | 1000 | 17.7 | 34.8 | 35 | 37 |
| Migraine with aura | 4446 | 89 | 23 | 41 | 25.2 | 34.4 | 20 | 38 |
| Graves' disease | 18020 | 196 | 45 | 361 | 13.4 | 33.4 | 39 | 39 |
| Celiac disease | 20718 | 213 | 55 | 399 | 12.9 | 32.2 | 40 | 40 |
| Myopia (pathological) | 3990 | 77 | 2 | 47 | 19.8 | 31.6 | 30 | 41 |
| Rheumatoid arthritis | 120346 | 1578 | 393 | 1599 | 16.4 | 29.7 | 36 | 42 |
| Pancreatic cancer | 73158 | 622 | 172 | 1351 | 10.9 | 29.3 | 43 | 43 |
| Primary biliary cirrhosis | 12347 | 93 | 25 | 146 | 9.6 | 21.4 | 44 | 44 |

*We used following search terms for ELI: ‘Disease name”, “Environment”, and “Lifestyle”. For Extended ELI we used ‘Disease name”, “Environment”, “Lifestyle”, and “Exposure” as search terms.
