# Supplementary material for: Allelic Spectra of Risk SNPs Are Different for Environment/Lifestyle Dependent versus Independent Diseases
Source: PLoS Genet. 2015 Jul 22;11(7):e1005371. doi: 10.1371/journal.pgen.1005371 (PMC4511800; doi:10.1371/journal.pgen.1005371)
Supplement: S1 Fig — (DOCX) [file pgen.1005371.s002.docx]

**Supplementary Materials**

**Figure s1.** Environment/Lifestyle Index (ELI) versus Extended Environment/Lifestyle Index (EELI)
